# High energy diet of beef cows during gestation promoted growth performance of calves by improving placental nutrients transport

**DOI:** 10.3389/fvets.2022.1053730

**Published:** 2022-11-24

**Authors:** Kun Kang, Lei Zeng, Jian Ma, Liyuan Shi, Rui Hu, Huawei Zou, Quanhui Peng, Lizhi Wang, Bai Xue, Zhisheng Wang

**Affiliations:** Low Carbon Breeding Cattle and Safety Production University Key Laboratory of Sichuan Province, Animal Nutrition Institute, Sichuan Agricultural University, Chengdu, China

**Keywords:** dietary energy, calf, growth performance, placenta, nutrient transporter

## Abstract

The aim of this study was to explore the effects of dietary energy level during gestation on growth performance and serum parameters in offspring using beef cattle as research objects. Additionally, the gene expressions associated with nutrients transport in the placenta were evaluated. Eighteen Simmental crossbred cows (body weight = 338.44 ± 16.03 kg and 760 ± 6 days of age) were randomly assigned to 3 dietary treatment groups: low energy (LE, metabolic energy = 8.76 MJ/kg), medium (ME, 9.47 MJ/kg) and high (HE, 10.18 MJ/kg). The dietary treatments were introduced from day 45 before expected date of parturition. The pre-experiment lasted for 15 days and formal experiment lasted for 30 days. Growth performance data and blood samples of calves were collected at birth and day 30 post-birth. The placental tissue was collected at parturition. The results indicated that the birth weight and average daily gain of calves in HE group were higher (*P* < 0.05) than those in LE group. After parturition, the serum contents of glucose, total protein, cortisol and leptin in neonatal calves were significantly increased (*P* < 0.05) with the elevation of dietary energy levels. At 30 days postpartum, the glucose, glutathione peroxidase, growth hormone, insulin-like growth factor 1 and leptin concentrations of HE group were significantly increased (*P* < 0.05) as compared with LE group, while the serum amyloid protein A displayed an opposite trend between two groups. With the increase of dietary energy concentration, placental mRNA expressions of vascular endothelial growth factor A, glucose transporter 1 and 3 were significantly up-regulated (*P* < 0.05). Furthermore, the amino acid transporter solute carrier family 38 member 1, hydroxysteroid 11-beta dehydrogenase 2, insulin-like growth factor 1 and 2 mRNA expressions of HE group were higher (*P* < 0.05) than those of LE and ME groups. In conclusion, the improved growth performance of calves from the high energy ration supplemented beef cows may be attributed to the increased placental nutrients transport, which may lead to the increased nutrient supply to the fetus.

## Introduction

Beef plays a vital role in the global food and nutrition security by providing high quality protein and key micronutrients for the humans (1). In China, with the development of economy, the consumption of beef has increased year by year. However, the beef production is limited and more and more beef cattle are imported in each year. According to statistics, the demand and import of beef were 9.3 and 2.2 million tons respectively in 2021. The rapidly rising demand of beef has become a problem that restricts the stable development of beef cattle industry in China. Due to the high mortality and morbidity and low growth rate of calves, the development of fattening cattle industry is faced with the problem of bovine shortage in recent years (2, 3). Calves are the future of beef cattle industry, and rearing healthy calves are essential to the production performance of fattening cattle in the future. At present, most researchers paid more attention to how to raise calves after birth (4, 5). In fact, late gestation is a critical period during which calf producers have substantial control of management of calves (6).

In ruminants breeding industry, the fetal growth retardation has been confirmed as an important factor that can affect the postnatal production performance (7). Previous study has reported that the healthy fetal development has long-term effects on immunity and organ function of animals, and fetal programming has been verified to affect the survival rate of calves and subsequent production performance (8). The concept of fetal programming is that maternal stimulation during critical period of fetal development has long-term effects on offspring (7). However, this depends on the degree of nutritional restriction, amplitude and duration of supplementation. Cows that experience nutritional restriction during the first two trimesters of gestation decreases the number of muscle fibers, while in the third trimester of gestation, cows malnutrition leads to lower birth weight and future growth rate of calves (9). On the contrary, higher nutrients intake by pregnant cows can induce the metabolic disorders of insulin, abnormal expression of genes related to the formation of adipocytes in the fetus and reduction of myogenesis, resulting in lower birth weight of calves (10, 11). As mentioned above, inconsistent results of maternal nutrition on the fetal development have been reported. Therefore, feeding appropriate nutritional level is important in fetal health.

A traditional view in dairy cow production during late gestation is to feed low energy ration due to concerning the postpartum metabolic disorders and milk production performance (12). However, low energy ration can reduce the birth weight and immune and antioxidant function of calves (13). In cattle and sheep production, previous studies have reported that maternal undernutrition during gestation causes some negative influence, such as the reduction of carcass composition and organ size of neonatal calves and lambs, and delay of subsequent puberty onset (14, 15). The placenta plays a critical role in modulating maternal-fetal resource allocation, thereby affecting fetal growth and long-term health of the offspring (16). The fetus exchanges substances with the mother mainly through the placental blood circulation system, obtaining nutrients and excreting metabolic wastes (17). As the interface between mother and fetus, the placenta can transport important nutrients, such as glucose (GLU), amino acid and fatty acid, from mother to fetus by specific transporters (18). Higher expressions of nutrient transporters in the placenta are beneficial for nutrients transport, which have important effects on the healthy development of fetus (19). Moreover, the up-regulated expressions of vascular endothelial growth factor A (VEGFA), insulin-like growth factor (IGF) and leptin (LEP) have significant influence on the placental angiogenesis and fetal birth weight (20, 21).

Although the nutritional physiology of newborn calf has been well-researched, the extent to which prenatal energy nutrition of cow affects the blood metabolites, immunity and antioxidant ability in neonatal calves, and placental gene expression associated with nutrients transport are yet to be further studied. Based on previous findings, we hypothesized that high maternal energy intake during late gestation could affect growth performance and immunity of calves by regulating the gene expression related to nutrients transport in the placenta. Therefore, the experiment was performed to investigate the effects of cows fed different dietary energy concentrations during the last 45 days prepartum on the growth performance and blood biochemical, immune, antioxidant and hormone parameters of calves. In addition, the nutrients transport gene expressions in the placenta were evaluated.

## Materials and methods

### Ethics statement

All experimental procedures involving animal care and management were authorized by the Institutional Animal Care and Use Committee of Sichuan Agricultural University (Chengdu, Sichuan, China).

### Experimental design and diet

This animal experiment was performed at a commercial beef cattle farm (Kunming, Yunan, China; altitude ~2,200 m; 25°30′ N latitude and 102°66′ E longitude). The current study was conducted from November (2020) to March (2021). A total of 18 healthy Simmental crossbred cattle in late gestation were used in this study. The selected cows [338.44 ± 16.03 kg of body weight (BW) and 760 ± 6 days of age] were randomly allocated to 3 groups with 6 cows in each group as follows: low energy (LE, metabolic energy = 8.76 MJ/kg), medium energy (ME, 9.47 MJ/kg) and high energy (HE, 10.18 MJ/kg).

In this research, the experimental diets were designed based on the NRC (22) recommendation for beef cattle. The ration of ME group was formulated according to the nutrient requirements of beef cattle at 350 kg and 9 months of gestation. Compared with ME group, the dietary energy levels of LE and HE groups were changed by 0.71 MJ/kg. The roughage-to-concentrate ratio of diets was adjusted to 60:40 and all the basal diets were isonitrogenous. The feed ingredients and nutrient levels of basal diet are shown in Table 1.

**Table 1:** Feed composition and nutrient levels of experimental rations (Dry matter basis).

**Items**	**Energy levels**		
	**LE**	**ME**	**HE**
**Ingredients, %**
Whole corn silage	25.00	35.00	40.00
Rice straw	35.00	25.00	20.00
Corn	6.00	14.00	20.50
Wheat bran	18.00	12.90	4.00
Cottonseed meal	1.00	2.75	1.00
Rapeseed meal	2.00	2.00	1.50
Soybean meal	1.00	1.50	5.45
Distillers dried grains with soluble	8.00	2.70	1.70
Active dry yeast	0.20	0.20	0.20
Fatty powder	0	0	1.50
CaCO_3_	0.40	0.55	0.45
CaHPO_4_	0.45	0.45	0.75
NaHCO_3_	0.45	0.45	0.45
NaCl	0.50	0.50	0.50
Premix^1^	2.00	2.00	2.00
**Nutrient levels, %**
Metabolic energy^2^, MJ/kg	8.76	9.47	10.18
Total digestible nutrient	63.43	66.01	69.74
Crude protein	10.16	10.17	10.18
Neutral detergent fiber	45.72	40.53	35.74
Acid detergent fiber	27.91	24.84	22.51
Ca	0.60	0.59	0.60
P	0.40	0.40	0.40

LE, low energy; ME, medium energy; HE, high energy.

1 The premix provided following per kilogram of experimental diet: VA 3000 IU, VD 500 IU, VE 50 IU, Cu (as copper sulfate) 10 mg, Fe (as ferrous sulfate) 50 mg, Mn (as manganese sulfate) 40 mg, Zn (as zinc sulfate) 30 mg, I (as potassium iodide) 0.5 mg, Se (as sodium selenite) 0.1 mg, Co (as cobalt chloride) 0.1 mg.

2 Metabolic energy was a calculated value; the other nutrient levels of the ration were measured values.

### Animal management

Before experiment, all cattle were marked with ear tags, and then housed in 18 pens with 1 cow in each pen. Each pen also had a fenced area used as an exercise ground for the cattle. All cows were fed a total mixed ration and regularly provided diets twice each day at 09:00 and 16:00. During the experiment, the animals had free access to water. A 15-day adaptive phase (−45 to −30 days relative to calving) was followed by 30 days of experimental period (−30 days to parturition).

The cows were moved to an individual delivery room that was carpeted with rice straw at 5 days prior to expected calving date. After parturition, cattle were transferred to an individual chute within 2 h and then milked *via* a transportable milking machine (Xulangte Machinery Co. Ltd., Zibo, Shandong, China). Colostrum yield was recorded and the immunoglobulin G (IgG) concentration was evaluated by a portable bovine colostrum detector (Yaming Instruments and Apparatus Co. Ltd., Xuzhou, Jiangsu, China).

The neonatal calves were processed after calving, mainly including navel disinfection, vaccination, earmark and weighing. Subsequently, calves were transferred to individual hutches. All the hutches were placed on rice straw that was renewed every 3 days. The calves' hutches were located at the fenced ares of their dams. The calves had free access to the hutches; however, the cows were not allowed to enter the hutches. All calves were fed with colostrum (10% of BW) within 1 h of birth from the respective dam. Then, the calves were cultivated by dam. After 7 days of birth, the calves were offered starter (21.16% crude protein and 14.22% neutral detergent fiber). Starter grain was provided once daily at 09:00 for *ad libitum* intake. Water was offered *ad libitum* during the experiment. In this study, the period of raising calves lasted for 30 days.

### Sample collection

On the first and thirtieth day after birth, the BW of all calves was measured *via* a digital scale before receiving milk, and the average daily gain (ADG) was obtained *via* the initial and final BW. In the meantime, the withers height, body length, cannon circumference and heart girth of calves were also determined. In addition, the blood samples were collected from the jugular vein of all calves by using evacuated tubes after weighing. Subsequently, blood samples were centrifuged at 3,000 rpm and 4°C for 15 min to separate serum. Serum samples were preserved in 1.5 mL sterile microtubes and stored at −20°C. After parturition, the placenta was washed by ice-cold sterile phosphate-buffered saline. Then, the placental samples from mid-portion were collected and snap-frozen in liquid nitrogen and stored at −80°C for quantitative real-time PCR analysis.

### Sample analysis

The frozen serum samples were thawed and thoroughly mixed. Then, serum samples were used to determine the biochemical parameters, including GLU, β-hydroxybutyric acid (BHBA), non-esterified fatty acid (NEFA), triglyceride (TG) and total protein (TP), *via* a automatic biochemical analyzer (BS-280, Mindray Bio-Medical Electronics Co. Ltd., Shenzhen, Guangdong, China). Moreover, the concentrations of IgG, haptoglobin (HP), ceruloplasmin (CER), cortisol (COR), serum amyloid protein A (SAA), interleukin 6 (IL-6), IL-10, tumor necrosis factor α (TNF-α), glutathione peroxidase (GSH-Px), superoxide dismutase (SOD), total antioxidant capacity (T-AOC), malondialdehyde (MDA), retinol (RET), tocopherol (TOC), growth hormone (GH), IGF-1, LEP, insulin (INS), and fibroblast growth factor 21 (FGF-21) were measured using commercial kits (Solarbio Science and Technology Co. Ltd., Beijing, China) according to the instructions.

Quantitative real-time PCR was used to quantitate the relative expressions of VEGFA, nitric oxide synthase 3 (NOS3), glucose transporter 1 (GLUT1), GLUT3, GLUT4, and amino acid transporter solute carrier family 38 member 1 (SLC38A1), SLC38A2, SLC38A4, fatty acid transport family protein 1 (FATP1), FATP4, fatty acid-binding protein 4 (FABP4), LEP, IGF-1, IGF-2, superoxide dismutase 1 (SOD1), catalase (CAT), GSH-Px, heat shock protein 70 (HSP70), and hydroxysteroid 11-beta dehydrogenase 2 (11β-HSD2) in the placental samples at the mRNA level. The cDNA was reversely transcribed from the extracted RNA, which was extracted from placental samples, using the cDNA Synthesis Kit (Sangon Biotechnology, Shanghai, China) reference to the descriptions. Quantitative real-time PCR was performed using the SYBR Green Kit (Sangon Biotechnology, Shanghai, China) and CFX96 Touch™ Real-Time PCR System (Bio-Rad Inc, Hercules, CA, USA) reference to the specifications. Each sample was processed in triplicate. The gene relative expressions were calculated using 2^−ΔΔCt^ method (23) with GAPDH as the housekeeping gene. The primers information of all genes which were designed by primer 5.0 software are presented in Supplementary Table S1.

### Statistical analysis

Before analysis, the normality and homogeneity of data were tested first. Subsequently, all data were analyzed by one-way ANOVA procedure of the SPSS statistical software (Version 20.0 for Windows; SPSS, Chicago, USA), with each animal as an experimental unit. The Duncan test was utilized to analyze the differences among three treatments. Data were presented as means and standard error of mean (SEM). A significance level was indicated at *P* < 0.05, and 0.05 ≤ *P* < 0.10 represented a tendency. Correlation analysis between differential genes and growth performance and serum parameters was conducted using GraphPad Prism software (version 7.0 for Windows; GraphPad Prism, San Diego, USA). *P*-value < 0.05 and the absolute value of correlation coefficient higher than 0.6 were deemed to be a significant correlation.

## Results

### Growth performance of calves

Effects of maternal dietary energy density on the growth performance of calves are shown in Table 2. At the first and thirtieth day after birth, the BW of calves in HE group was higher (*P* < 0.05) than that in LE group. Compared with LE group, the ADG of HE group was increased by 30.16% (*P* < 0.05). During the experiment, no significant difference (*P* > 0.05) of withers height, body length, cannon circumference and heart girth was found among three groups.

**Table 2:** Effects of maternal dietary energy concentration on the growth performance of calves.

**Items**	**Groups**			**SEM**	***P*-value**
	**LE**	**ME**	**HE**		
Birth weight, kg	26.83^b^	29.43^ab^	32.38^a^	1.22	0.02
Final weight, kg	45.62^b^	50.07^ab^	56.93^a^	2.73	0.03
ADG, kg	0.63^b^	0.69^ab^	0.82^a^	0.07	0.03
**Body measurement (birth, cm)**					
Withers height	67.00	70.00	71.00	2.02	0.19
Body length	56.67	60.17	62.67	2.04	0.14
Cannon circumference	11.00	11.33	11.58	0.33	0.48
Heart girth	76.09	76.58	77.67	1.66	0.47
**Body measurement (30 days of age, cm)**					
Withers height	75.17	77.50	79.83	1.78	0.21
Body length	71.17	71.17	74.50	1.26	0.13
Cannon circumference	12.17	12.12	12.50	0.36	0.72
Heart girth	84.33	85.06	86.11	1.20	0.24

LE, low energy; ME, medium energy; HE, high energy; BW, body weight; ADG, average daily gain; SEM, standard error of the mean.

In the same row, values with different small letter mean significant difference (P < 0.05).

### Serum biochemical index of calves

As shown in Table 3, on day 1, the concentrations of BHBA, NEFA, TG and IgG were similar (*P* > 0.05) among three groups. However, the serum contents of GLU and TP were significantly increased (*P* < 0.05) with the rise of dietary energy levels. On day 30, the serum GLU content of calves in ME and HE groups was higher (*P* < 0.05) than that in LE group. No obvious difference (*P* > 0.05) of BHBA, NEFA, TG, TP, and IgG was observed among all groups.

**Table 3:** Effects of maternal dietary energy concentration on the serum biochemical indexes of calves.

**Items**	**Groups**			**SEM**	***P*-value**
	**LE**	**ME**	**HE**		
**Day 1**					
GLU, mmol/L	2.73^c^	3.17^b^	4.04^a^	0.09	< 0.01
BHBA, mmol/L	0.46	0.40	0.38	0.03	0.14
NEFA, mmol/L	0.34	0.32	0.29	0.01	0.15
TG, mmol/L	0.35	0.36	0.36	0.02	0.68
TP, g/L	36.97^c^	38.53^b^	39.92^a^	0.39	< 0.01
IgG, g/L	0.59	0.56	0.54	0.02	0.19
**Day 30**					
GLU, mmol/L	4.60^b^	5.35^a^	5.48^a^	0.06	< 0.01
BHBA, mmol/L	0.05	0.05	0.04	0.00	0.12
NEFA, mmol/L	0.20	0.22	0.20	0.01	0.34
TG, mmol/L	0.29	0.30	0.30	0.02	0.46
TP, g/L	38.69	39.07	39.46	0.41	0.43
IgG, g/L	14.38	14.29	13.97	0.23	0.14

LE, low energy; ME, medium energy; HE, high energy; GLU, glucose; BHBA, β-hydroxybutyric acid; NEFA, non-esterified fatty acid; TG, triglyceride; TP, total protein; IgG, immunoglobulin G; SEM, standard error of the mean.

In the same row, values with different small letter mean significant difference (P < 0.05).

### Serum inflammatory index of calves

The concentrations of HP, CER, and SAA in serum did not show significant difference (*P* > 0.05) among three groups on day 1 (Table 4). As dietary energy levels rise, the serum COR content was significantly increased (*P* < 0.05). Additionally, the IL-6 and TNF-α concentrations in serum of HE group were higher (*P* < 0.05) than those of LE group, whereas the IL-10 content exhibited an opposite trend between two groups. On day 30, the SAA content was significantly decreased (*P* < 0.05) with the increase of dietary energy levels. No significant difference (*P* > 0.05) of other parameters was found among three groups.

**Table 4:** Effects of maternal dietary energy concentration on the serum inflammatory indexes of calves.

**Items**	**Groups**			**SEM**	***P*-value**
	**LE**	**ME**	**HE**		
**Day 1**					
HP, mg/mL	0.44	0.44	0.46	0.03	0.86
CER, umol/L	0.58	0.57	0.63	0.03	0.43
COR, ng/mL	40.69^c^	42.69^b^	47.00^a^	0.40	< 0.01
SAA, μg/mL	53.45	55.63	59.60	2.10	0.15
IL-6, ng/mL	0.27^b^	0.34^a^	0.38^a^	0.02	0.01
IL-10, 10^−1^ ng/mL	0.77^a^	0.74^ab^	0.66^b^	0.03	0.03
TNF-α, ng/mL	0.17^b^	0.18^b^	0.22^a^	0.01	0.02
**Day 30**					
HP, mg/mL	0.29	0.28	0.27	0.01	0.58
CER, umol/L	2.34	2.24	2.21	0.07	0.38
COR, ng/mL	17.35	17.95	17.47	0.32	0.41
SAA, μg/mL	146.30^a^	136.17^b^	129.9^c^	1.97	< 0.01
IL-6, ng/mL	0.19	0.19	0.18	0.01	0.79
IL-10, 10^−1^ ng/mL	0.98	0.97	0.95	0.03	0.69
TNF-α, ng/mL	0.10	0.11	0.11	0.01	0.60

LE, low energy; ME, medium energy; HE, high energy; HP, haptoglobin; CER, ceruloplasmin; COR, cortisol; SAA, serum amyloid protein A; IL-6, interleukin-6; IL-10, interleukin-10; TNF-α, tumor necrosis factor α; SEM, standard error of the mean.

In the same row, values with different small letter mean significant difference (P < 0.05).

### Serum antioxidant index of calves

On day 1, the GSH-Px and RET concentrations of LE and ME groups were significantly increased (*P* < 0.05) as compared with HE group (Table 5). The serum T-AOC activity of LE group was slightly higher (*P* = 0.06) than that of ME group. The SOD, MDA and TOC concentrations in serum of calves were similar (*P* > 0.05) among all groups. On day 30, the serum concentrations of SOD and T-AOC were not different (*P* > 0.05) among all groups. However, compared with LE and ME groups, the GSH-Px activity of HE group was significantly increased (*P* < 0.05). With the increase of dietary energy levels, the RET and TOC contents were markedly elevated (*P* < 0.05). Moreover, the MDA activity in ME group tended to be higher (*P* = 0.08) than that in LE group.

**Table 5:** Effects of maternal dietary energy concentration on the serum antioxidant indexes of calves.

**Items**	**Groups**			**SEM**	***P*-value**
	**LE**	**ME**	**HE**		
**Day 1**					
GSH-Px, U/mL	175.97^a^	176.69^a^	166.07^b^	2.44	0.01
SOD, U/mL	90.63	86.44	87.34	2.22	0.39
T-AOC, U/mL	22.78	21.36	20.10	0.73	0.06
MDA, nmol/mL	7.79	7.65	7.95	0.12	0.20
RET, μg/dL	8.31^a^	8.21^a^	7.45^b^	0.07	< 0.01
TOC, μg/mL	0.32	0.31	0.33	0.01	0.59
**Day 30**					
GSH-Px, U/mL	149.48^b^	153.66^b^	165.47^a^	3.07	< 0.01
SOD, U/mL	94.62	97.60	95.91	2.03	0.59
T-AOC, U/mL	23.00	21.62	23.48	1.30	0.58
MDA, nmol/mL	5.51	6.02	5.85	0.15	0.08
RET, μg/dL	28.23^c^	33.11^b^	35.08^a^	0.20	< 0.01
TOC, μg/mL	2.31^c^	2.41^b^	2.97^a^	0.03	< 0.01

LE, low energy; ME, medium energy; HE, high energy; GSH-Px, glutathione peroxidase; SOD, superoxide dismutase; T-AOC, total antioxidant capacity; MDA, malondialdehyde; RET, retinol; TOC, tocopherol; SEM, standard error of the mean.

In the same row, values with different small letter mean significant difference (P < 0.05).

### Serum hormone and growth factor of calves

Compared with HE group, the GH concentration of LE and ME groups was significantly increased (*P* < 0.05), while the FGF-21 displayed a contrary tendency on day 1 (Table 6). LE group had highest serum INS content that was higher (*P* < 0.05) than ME and HE groups. However, the IGF-1 and LEP contents of HE group were higher (*P* < 0.05) than those of LE group. On day 30, no obvious difference (*P* > 0.05) of INS and FGF-21 was found among three groups. The serum GH and IGF-1 concentrations of HE group were higher (*P* < 0.05) than those of LE and ME groups. Similarly, the LEP content of ME and HE groups was increased (*P* < 0.05) as compared with LE group.

**Table 6:** Effects of maternal dietary energy concentration on the serum hormone and growth factor of calves.

**Items**	**Groups**			**SEM**	***P*-value**
	**LE**	**ME**	**HE**		
**Day 1**					
GH, ng/mL	5.11^a^	5.26^a^	4.23^b^	0.21	< 0.01
IGF-1, ng/mL	150.91^b^	171.08^a^	169.31^a^	2.81	0.04
LEP, ng/mL	0.80^c^	0.85^b^	0.93^a^	0.02	< 0.01
INS, ug/L	1.01^a^	0.91^b^	0.88^b^	0.03	0.02
FGF-21, ug/L	1.35^b^	1.34^b^	1.42^a^	0.02	0.02
**Day 30**					
GH, ng/mL	10.42^b^	10.68^b^	12.02^a^	0.18	< 0.01
IGF-1, ng/mL	222.76^b^	220.75^b^	254.84^a^	4.94	< 0.01
LEP, ng/mL	2.15^b^	2.32^a^	2.37^a^	0.06	0.02
INS, ug/L	1.06	1.08	1.05	0.04	0.89
FGF-21, ug/L	0.93	0.98	1.04	0.04	0.20

LE, low energy; ME, medium energy; HE, high energy; GH, growth hormone; IGF-1, insulin-like growth factor 1; LEP, leptin; INS, insulin; FGF-21, fibroblast growth factor 21; SEM, standard error of the mean.

In the same row, values with different small letter mean significant difference (P < 0.05).

### Gene mRNA expression in the placenta

**Figure 1:**
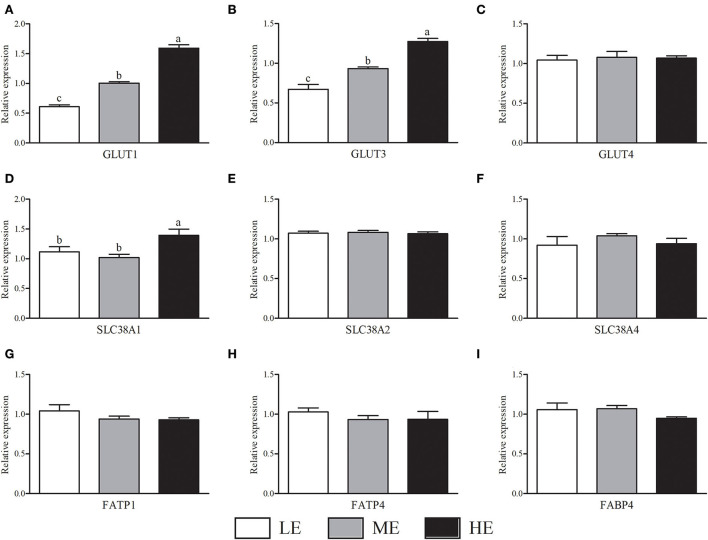
Effects of maternal dietary energy concentration on the mRNA expressions of glucose **(A–C)**, amino acid **(D–F)**, and fatty acid **(G–I)** transporters in the placenta. LE, low energy (metabolic energy = 8.76 MJ/kg); ME, medium energy (metabolic energy = 9.47 MJ/kg); HE, high energy (metabolic energy = 10.18 MJ/kg); GLUT1, glucose transporter 1; GLUT3, glucose transporter 3; GLUT4, glucose transporter 4; SLC38A1, amino acid transporter solute carrier family 38 member 1; SLC38A2, amino acid transporter solute carrier family 38 member 2; SLC38A4, amino acid transporter solute carrier family 38 member 4; FATP1, fatty acid transport family protein 1; FATP4, fatty acid transport family protein 4; FABP4, fatty acid-binding protein 4. Means in the columns without a common small letter differ (*P* < 0.05).

Notably, with the increase of dietary energy concentration, the mRNA expressions of GLUT1 (Figure 1A) and GLUT3 (Figure 1B) were significantly up-regulated (*P* < 0.05). Additionally, the SLC38A1 mRNA expression (Figure 1D) of HE group was higher (*P* < 0.05) than that of LE and ME groups. There was no significant difference (*P* > 0.05) of GLUT4, SLC38A2, SLC38A4, FATP1, FATP4, and FABP4 mRNA expressions in the placenta among three groups (Figure 1).

**Figure 2:**
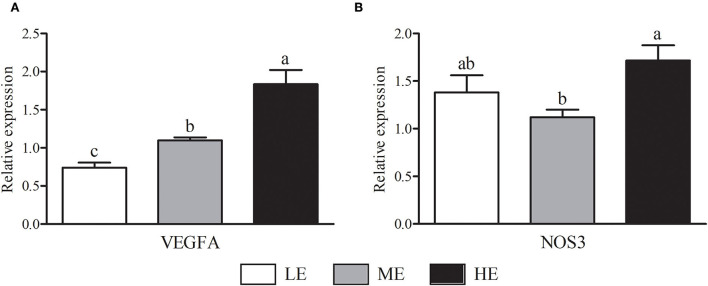
Effects of maternal dietary energy concentration on the mRNA expressions of VEGFA **(A)** and NOS3 **(B)** in the placenta. LE, low energy (metabolic energy = 8.76 MJ/kg); ME, medium energy (metabolic energy = 9.47 MJ/kg); HE, high energy (metabolic energy = 10.18 MJ/kg); VEGFA, vascular endothelial growth factor A; NOS3, nitric oxide synthase 3. Means in the columns without a common small letter differ (*P* < 0.05).

**Figure 3:**
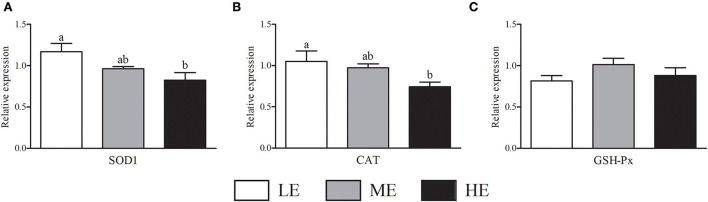
Effects of maternal dietary energy concentration on the mRNA expressions of SOD1 **(A)**, CAT **(B)**, and GSH-Px **(C)** in the placenta. LE, low energy (metabolic energy = 8.76 MJ/kg); ME, medium energy (metabolic energy = 9.47 MJ/kg); HE, high energy (metabolic energy = 10.18 MJ/kg); SOD1, superoxide dismutase 1; CAT, catalase; GSH-Px, glutathione peroxidase. Means in the columns without a common small letter differ (*P* < 0.05).

**Figure 4:**
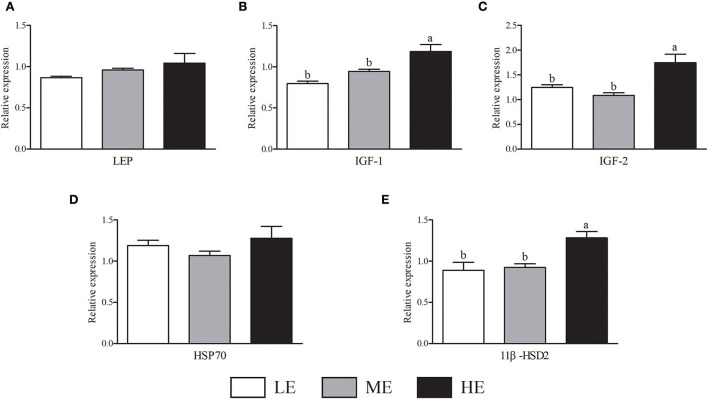
Effects of maternal dietary energy concentration on the mRNA expressions of LEP **(A)**, IGF-1 **(B)**, IGF-2 **(C)**, HSP70 **(D)**, and 11β-HSD2 **(E)** in the placenta. LE, low energy (metabolic energy = 8.76 MJ/kg); ME, medium energy (metabolic energy = 9.47 MJ/kg); HE, high energy (metabolic energy = 10.18 MJ/kg); LEP, leptin; IGF-1, insulin-like growth factor 1; IGF-2, insulin-like growth factor 2; HSP70, heat shock protein 70; 11β-HSD2, hydroxysteroid 11-beta dehydrogenase 2. Means in the columns without a common small letter differ (*P* < 0.05).

As shown in Figure 2, HE group had maximum VEGFA mRNA expression that was higher (*P* < 0.05) than LE and ME groups. Compared with ME group, the mRNA expression of NOS3 in HE group was increased by 52.68% (*P* < 0.05). In Figure 3, the SOD1 and CAT mRNA expressions of LE group were higher (*P* < 0.05) than those of HE group. However, the expression of GSH-Px (Figure 3C) was similar (*P* > 0.05) among all groups. Overall, higher dietary energy level increased (*P* < 0.05) the IGF-1 (Figure 4B), IGF-2 (Figure 4C), and 11β-HSD2 (Figure 4E) mRNA expressions. No obvious difference (*P* > 0.05) of LEP (Figure 4A) and HSP70 (Figure 4D) was observed among three groups.

### Associations between gene expression and growth performance and serum parameters

**Figure 5:**
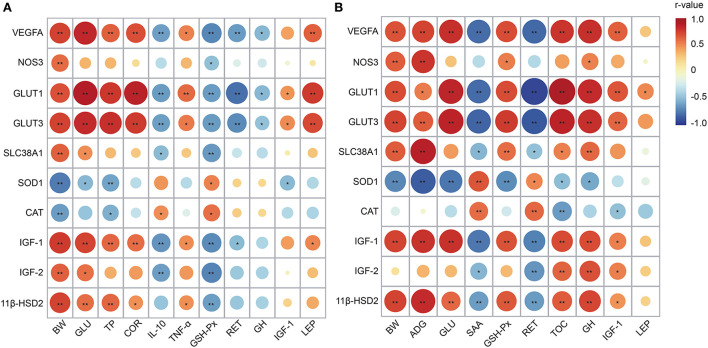
Correlation analysis between placental genes and growth performance and serum parameters collected on day 1 **(A)** and 30 **(B)**. The circle in red color represents a positive correlation (*r* > 0.6 and *P* < 0.05), and the circle in blue color represents a negative correlation (*r* < −0.6 and *P* < 0.05). The circle with larger size and darker color indicates a higher correlation. BW, body weight; ADG, average daily gain; GLU, glucose; TP, total protein; COR, cortisol; IL-10, interleukin-10; TNF-α, tumor necrosis factor α; SAA, serum amyloid protein A; GSH-Px, glutathione peroxidase; RET, retinol; TOC, tocopherol; GH, growth hormone; IGF-1, insulin-like growth factor 1; LEP, leptin; VEGFA, vascular endothelial growth factor A; NOS3, nitric oxide synthase 3; GLUT1, glucose transporter 1; GLUT3, glucose transporter 3; SLC38A1, amino acid transporter solute carrier family 38 member 1; SOD1, superoxide dismutase 1; CAT, catalase; IGF-2, insulin-like growth factor 2; 11β-HSD2, hydroxysteroid 11-beta dehydrogenase 2.

Correlation analysis revealed that the genes, including VEGFA, NOS3, GLUT1, GLUT3, SLC38A1, IGF-1, IGF-2, and 11β-HSD2, were positively correlated with birth weight (*r* ranged from 0.603 to 0.772, *P* < 0.05) (Figure 5A). The VEGFA, GLUT1, GLUT3, and IGF-1 mRNA expressions had positive correlations with serum GLU, TP, and COR concentrations collected on day 1 (*r* ranged from 0.600 to 0.898, *P* < 0.05). However, an opposite relationship was found between those genes and IL-10 and GSH-Px (*r* ranged from −0.713 to −0.620, *P* < 0.05). Moreover, the VEGFA, GLUT1, and GLUT3 were negatively correlated with RET (*r* ranged from −0.796 to −0.632, *P* < 0.05) and positively correlated with LEP (*r* ranged from 0.638 to 0.769, *P* < 0.05).

As shown in Figure 5B, the mRNA expressions of VEGFA, NOS3, GLUT3, SLC38A1, IGF-1, and 11β-HSD2 were positively correlated with BW and ADG collected on day 30 (*r* ranged from 0.647 to 0.882, *P* < 0.05). An opposite relationship was observed between SOD1 and those parameters (*r* ranged from −0.858 to −0.705, *P* < 0.05). The VEGFA, GLUT1, GLUT3, IGF-1, and 11β-HSD2 had positive correlations with serum GLU, GSH-Px, TOC, and GH contents (*r* ranged from 0.652 to 0.918, *P* < 0.05). However, there was a negative correlation between those genes and SAA and RET (*R* ranged from −0.938 to −0.627, *P* < 0.05). Besides, the VEGFA, GLUT1, and GLUT3 displayed positive correlations with IGF-1 (*r* ranged from 0.626 to 0.677, *P* < 0.05).

## Discussion

The prenatal development has important impact on the healthy growth and production performance of cattle throughout postnatal life. Maternal nutrition, which provides amino acid, vitamin, GLU and fatty acid to the fetus by placenta, plays an important role in the growth and development of fetus (24). Late gestation is a critical period because the growth and development of fetus occur the last 2 trimesters of gestation (25). Thus, in this period, cows undergo an increased nutritional requirements to maintain body health and fetal development. Inadequate maternal nutrition during gestation seriously affects the fetal development since the changes of maternal metabolic status damages provision of fetal nutrients, which would compromise postnatal growth and health of fetus (26). Our previous study found that maternal malnutrition reduced the birth weight and increased morbidity of calves (27). In the current study, higher dietary energy density of beef cows increased the birth weight of calves, suggesting that the fetus could obtain more nutrients from the cows, which was conducive to promoting growth. A recent study reported that calves born to cows fed the high energy ration during gestation were significantly heavier than those born to cows fed low energy ration at birth (28), which were in accordance with our results.

Our results indicated that the BW at 30 days postpartum and ADG of HE group were higher than those of LE group, indicating that higher prenatal nutrition had long-term effects on growth rate of calves. Consistent with our findings, a study used beef cows as research objects and found that the calves of cows fed high energy diet were significantly heavier than those of the low energy diet-fed cows at 3 weeks postpartum (29). To a certain extent, the body measurements can be used to evaluate the development situation of calves (30). In our trial, the body measurements were similar among all groups. Inconsistent with our study, Gao et al. (13) reported that higher prepartum maternal energy increased withers height, body length and thoracic girth of Holstein calves. The reason may be that the birth weight of Simmental crossbred calves was less than Holstein calves, and the difference of body measurements in Simmental crossbred calves was relatively lower.

Feeding low energy diet is commonly used in dairy cows production because of considering postpartum disease and milking performance (12). However, lower maternal energy intake decreases the birth weight and immunity of calves. In beef cattle production, higher birth weight of calves is favorable to future productivity (31). On the other hand, high energy diet during late gestation can induce difficult labor. In this study, the dietary energy levels were within the range of 8.76–10.18 MJ/kg. Although the difference of energy level was relatively small (0.71 MJ/kg), we observed that the rate of dystocia in HE group was 33.33%. Thus, in production, the dietary energy level should be considered. According to our results, a reasonable rise of dietary energy level could increase the birth weight and postpartum growth of calves. Generally, the better birth weight of calves is associated with improved nutrients supply before parturition. Therefore, we performed the following study to explore the effects of dietary energy level of beef cows on nutrients supply by collecting blood samples of calves and placental tissue.

Blood biochemical parameters, which are associated with metabolic status of body, can be used to reflect the health of animals (32). As a key index of protein metabolism, the serum content of TP is commonly used to evaluate whether the calves has achieved passive immunity. In the present study, no obvious difference of IgG was found among three groups, but higher dietary energy increased the serum TP content. The results indicated that calves in HE group had higher ability to produce immunoglobulin. Serum GLU concentration is an important parameter that can reflect the energy metabolism of animals. In dairy calves, a previous research reported that increasing prepartum maternal energy density could increase the serum GLU content of neonatal calves (13). Consistent with previous study, we found that higher dietary energy level of beef cows significantly increased the GLU content in serum of calves. Increased GLU content was conducive to promoting growth of calves, which matched to growth performance data. The possible reason is that dietary energy improves the maternal glycometabolism and promotes GLU transfer by the placenta, which result in increased GLU content in blood of calves.

As important inflammatory mediators, the IL-6 and TNF-α are closely related to body inflammation. In addition, IL-10 plays an essential role in the synthesis of pro-inflammatory cytokines, then relieves the damage of inflammatory response to the body (33). In our study, the HE group exhibited higher contents of IL-6 and TNF-α and lower content of IL-10 in serum as compared to LE group, suggesting that the calves in HE group may exist inflammatory response after parturition. These results may be attributed to difficult labor in beef cows fed high energy diet. At 30 days postpartum, no significant difference of these parameters was found among all groups, which indicated that all the calves were in a healthy state. As a glucocorticoid, COR is released after an acute-phase response and can regulate immune response (34). In calves, the blood COR concentration peak after parturition (35). The present study showed that with the elevation of maternal dietary energy level, the serum COR content was significantly increased. A previous study has reported that COR may induce acute-phase response that can damage the innate and humoral immune reaction (36). High dietary energy diet resulted in difficult labor of beef cows and then induced inflammatory response of neonatal calves. However, as the days of age increasing, the inflammatory response was gradually relieved.

In general, the reactive oxygen free radical will be produced during the development and metabolism of animals. The accumulated free radicals can impair the structure and function of cells, resulting in oxidative stress (5). Moreover, stress reaction, such as the change of living environment and difficult labor, can induce oxidative stress of calves (37). Maternal energy level affected the antioxidant ability of neonatal calves. A previous study found that higher maternal dietary energy could enhance the activity of serum GSH-Px, SOD and T-AOC in neonatal calves (28). However, in our study, the serum GSH-Px and T-AOC activities of neonatal calves in HE group were lower than LE group. GSH-Px can suppress lipid peroxidation *via* eliminating excessive free radicals in the body. T-AOC reflects the antioxidative ability of the body's defense system (30). Our results indicated that the neonatal calves of HE group existed oxidative stress, which may be related to mogitocia caused by higher dietary energy level. The RET and TOC have important effects on the regulation of oxidative stress (38, 39). In the current study, the serum RET concentration of HE group at birth was lower than LE group, while the RET and TOC concentrations displayed an opposite trend between two groups on day 30. Likewise, the GSH-Px activity of HE group was significantly increased as compared with LE group. After a period of growth, calves born to cows fed the high energy ration during late gestation showed improved antioxidant ability, suggesting that higher maternal energy level had long-term effect on antioxidant ability of calves.

As a peptide hormone, the GH can regulate protein synthesis, fatty and mineral metabolism, and plays a key role in animals' growth and development. IGF-1 is an active protein polypeptide that is necessary in the physiological process of GH action (40). In sheep, an early study reported that nutrient restriction of singleton pregnancies could lead to elevated GH concentration in fetus (41). Similarly, in our study, the serum GH concentration of HE group was lower than LE and ME groups after birth. Low nutrient levels required more GH to regulate energy metabolism and maintain body health, which might explain why LE group had higher GH levels. At 30 days of age, the HE group displayed higher GH concentration. Likewise, the IGF-1 concentration of HE group was higher than LE group, which was beneficial for growth of calves. When animals experience malnutrition or reduced body fat, the serum LEP decreases significantly, then stimulates the ability to ingest nutrients and reduces body energy expenditure (42). Our results showed that higher dietary maternal energy could increase the serum LEP content of calves, indicating that higher dietary energy level was beneficial for growth and development of calves. The FGF-21 can promote GLU inhalation by adipocytes, which has important effects on the lipid and carbohydrate metabolism (43). In the current study, higher maternal energy intake increased serum FGF-21 concentration of neonatal calves. The increase of serum FGF-21 in calves may be partly attributed to the response of fetus to high energy nutrients from maternal body (44). However, the specific regulation mechanism still needs elucidation.

As a channel, the placenta acts as a vital role in the fetal growth and development by transporting nutrients and oxygen from mothers. Generally, the fetal development is mainly affected by maternal nutrient availability and placental transport efficiency (45). The healthy development of placental angiogenesis is essential for transporting nutrients from mother to fetus (46). In this experiment, VEGFA and NOS3, key angiogenesis factors (47), were expressed higher in the placenta of beef cows receiving high energy ration than in the LE cows, suggesting that placental angiogenesis may be enhanced by high energy ration. This effect of high energy diet contributes to promoting the transfer of nutrients and oxygen to the fetus, then improving the future growth and development of calves as it was been observed that VEGFA and NOS3 expressions were positively correlated with BW, ADG, serum GLU, and TP contents.

Fetal access to maternal nutrients requires the participation of nutrient transporters in the placenta. As an important energy source, GLU is essential for healthy growth of fetus. The transport of GLU in the placenta is regulated by facilitated diffusion through GLU transporters (e.g., GLUT1, GLUT3, and GLUT4) (47). In our study, with the elevation of dietary energy levels, the mRNA expressions of GLUT1 and GLUT3 were significantly up-regulated, which indicated that calves in HE group could obtain more GLU to promote growth. The GLUT1 is mainly expressed in basal membrane of placenta, while GLUT3 is located in microvillus membrane (48). The up-regulated expressions of GLUT1 and GLUT3 may be related to the increased placental angiogenesis. In addition to GLU, the transport of amino acid and fatty acid through placenta is also important for fetal growth. We found that the SLC38A1 expression of HE group was significantly increased as compared to LE and ME groups, indicating that higher dietary energy concentration could improve the utilization of amino acid in fetus. Batistel et al. (49) found that the increased birth weight of neonatal calves was strongly related to the up-regulated mRNA expression of genes encoding amino acid, GLU and fatty acid transporters in the placenta. Our experiment showed that the expressions of GLU and amino acid transporters displayed positive correlations with growth performance of calves, which were in accordance with previous results.

During pregnancy, insulin-like growth factors in the placenta are involved in the regulation of GLU and amino acid transport, as well as glycogen reserve, and are associated with fetal birth weight. A previous study reported that feeding high fat diet of mice can up-regulate the placental mRNA expression of IGF-2, which promotes the growth of offspring (50). Consistent with previous research, our results showed that high energy diet significantly increased the IGF-1 and IGF-2 mRNA expressions in the placenta, suggesting that the fetus obtained more nutrients from cows as reflected in the positive correlations between IGF-1 expression and serum nutrients contents. As a metabolic enzyme of glucocorticoid secreted by the placenta, the 11β-HSD2 prevents excessive maternal glucocorticoid from entering the fetus and avoids the harm of fetal physical decline in the future (51). In the current study, high energy diet improved 11β-HSD2 expression in the placenta, which had protective effects for future growth of calves as shown in the positive correlation between 11β-HSD2 and ADG. A study in piglets found that the placenta for low birth weight neonate was vulnerable to oxidative stress (46). However, in our study, the HE group showed lower levels of SOD1 and CAT, indicating that the dietary energy concentration should be controlled within a certain range. In the future, the mechanism of high energy diet regulating the expression of placental nutrient transporters deserves in-depth investigation.

## Conclusion

The results from our study showed that appropriate increase of dietary energy concentration (0.71 MJ/kg) in Simmental crossbred beef cows during late gestation improves the growth performance of calves. This beneficial effect of high dietary energy level may be attributed to the increased supply of nutrients to fetus from mother, mediated by up-regulated genes expression associated with nutrients transport in the placenta.

## Data availability statement

The original contributions presented in the study are included in the article/Supplementary material, further inquiries can be directed to the corresponding author.

## Ethics statement

The animal study was reviewed and approved by Institutional Animal Care and Use Committee of Sichuan Agricultural University.

## Author contributions

KK, HZ, and ZW conceived and designed the research. KK, LZ, LS, QP, LW, and BX performed the animal experiment and samples analysis. KK, JM, and RH analyzed the data. KK wrote the original manuscript. KK, JM, RH, and ZW reviewed the manuscript. All authors read and approved the final manuscript.

## Funding

This study was supported by the Sichuan Science and Technology Program (2021YFYZ0001).

## Conflict of interest

The authors declare that the research was conducted in the absence of any commercial or financial relationships that could be construed as a potential conflict of interest.

## Publisher's note

All claims expressed in this article are solely those of the authors and do not necessarily represent those of their affiliated organizations, or those of the publisher, the editors and the reviewers. Any product that may be evaluated in this article, or claim that may be made by its manufacturer, is not guaranteed or endorsed by the publisher.

## References

[1] Vahmani P Ponnampalam EN Kraft J Mapiye C Bermingham EN Watkins PJ. Bioactivity and health effects of ruminant meat lipids. Invited review Meat Sci. (2020) 165:108114. 10.1016/j.meatsci.2020.108114 32272342

[2] Du R Jiao S Dai Y An J Lv J Yan X. Probiotic *Bacillus amyloliquefaciens* C-1 improves growth performance, stimulates GH/IGF-1, and regulates the gut microbiota of growth-retarded beef calves. Front Microbiol. (2018) 9:2006. 10.3389/fmicb.2018.02006 30210477 PMC6120984

[3] Ma J Shah AM Wang ZS Hu R Zou HW Wang XY. Dietary supplementation with glutamine improves gastrointestinal barrier function and promotes compensatory growth of growth-retarded yaks. Animal. (2021) 15:100108. 10.1016/j.animal.2020.100108 33712211

[4] Sarah S Asako K Liane H Laurenz S Saskia K Karlheinz S. Weaning age influences indicators of rumen function and development in female Holstein calves. BMC Vet Res. (2022) 18:102. 10.1186/s12917-022-03163-1 35300681 PMC8928593

[5] Ma J Wang C Wang Z Cao G Hu R Wang X. Active dry yeast supplementation improves the growth performance, rumen fermentation, and immune response of weaned beef calves. Anim Nutr. (2021) 7:1352–9. 10.1016/j.aninu.2021.06.006 34786508 PMC8577086

[6] Wilson TB Schroeder AR Ireland FA Faulkner DB Shike DW. Effects of late gestation distillers grains supplementation on fall-calving beef cow performance and steer calf growth and carcass characteristics. J Anim Sci. (2015) 93:4843–51. 10.2527/jas.2015-9228 26523577

[7] Sartori ED Sessim AG Brutti DD Lopes JF McManus CM Barcellos JOJ. Fetal programming in sheep: effects on pre- and postnatal development in lambs. J Anim Sci. (2020) 98:skaa294. 10.1093/jas/skaa294 32894763 PMC7521827

[8] Rehfeldt C Te Pas MFW Wimmers K Brameld JM Nissen PM Berri C. Advances in research on the prenatal development of skeletal muscle in animals in relation to the quality of muscle-based food. I Regulation of myogenesis and environmental impact. Animal. (2011) 5:703–17. 10.1017/S1751731110002089 22439993

[9] Gutiérrez V Espasandín AC Machado P Bielli A Genovese P Carriquiry M. Effects of calf early nutrition on muscle fiber characteristics and gene expression. Livest Sci. (2014) 167:408–16. 10.1016/j.livsci.2014.07.01020525929

[10] Radunz AE Fluharty FL Relling AE Felix TL Shoup LM Zerby HN. Prepartum dietary energy source fed to beef cows: II. Effects on progeny postnatal growth, glucose tolerance, and carcass composition. J Anim Sci. (2012) 90:4962–74. 10.2527/jas.2012-5098 22952375

[11] Wilson TB Faulkner DB Shike DW. Influence of prepartum dietary energy on beef cow performance and calf growth and carcass characteristics. Livest Sci. (2016) 184:21–7. 10.1016/j.livsci.2015.12.00416908645

[12] Janovick NA Boisclair YR Drackley JK. Prepartum dietary energy intake affects metabolism and health during the periparturient period in primiparous and multiparous Holstein cows. J Dairy Sci. (2011) 94:1385–400. 10.3168/jds.2010-3303 21338804

[13] Gao F Liu YC Zhang ZH Zhang CZ Su HW Li SL. Effect of prepartum maternal energy density on the growth performance, immunity, and antioxidation capability of neonatal calves. J Dairy Sci. (2012) 95:4510–8. 10.3168/jds.2011-5087 22818465

[14] Long NM Tousley CB Underwood KR Paisley SI Means WJ Hess BW. Effects of early- to mid-gestational undernutrition with or without protein supplementation on offspring growth, carcass characteristics, and adipocyte size in beef cattle. J Anim Sci. (2012) 90:197–206. 10.2527/jas.2011-4237 21908644

[15] Knight MI Butler KL Slocombe LL Linden NP Raeside MC Burnett VF. Reducing the level of nutrition of twin-bearing ewes during mid to late pregnancy produces leaner prime lambs at slaughter. Animal. (2020) 14:864–72. 10.1017/S1751731119002271 31610822

[16] Redifer CA Duncan NB Meyer AM. Factors affecting placental size in beef cattle: maternal and fetal influences. Theriogenology. (2021) 174:149–59. 10.1016/j.theriogenology.2021.08.015 34454320

[17] Newbern D Freemark M. Placental hormones and the control of maternal metabolism and fetal growth. Curr Opin Endocrinol. (2011) 18:409–16. 10.1097/MED.0b013e32834c800d 21986512

[18] Gu F Jiang L Xie L Wang D Zhao F Liu J. Supplementing N-carbamoylglutamate in late gestation increases newborn calf weight by enhanced placental expression of mTOR and angiogenesis factor genes in dairy cows. Anim Nutr. (2021) 7:981–8. 10.1016/j.aninu.2021.05.007 34738028 PMC8551415

[19] Gaccioli F Lager S. Placental nutrient transport and intrauterine growth restriction. Front Physiol. (2016) 7:40. 10.3389/fphys.2016.00040 26909042 PMC4754577

[20] Guimarães GC Alves LA Betarelli RP Guimarães CSO Helmo FR Pereira Júnior CD. Expression of vascular endothelial growth factor (VEGF) and factor VIII in the gilt placenta and its relation to fetal development. Theriogenology. (2017) 92:63–8. 10.1016/j.theriogenology.2017.01.002 28237345

[21] Farley DM Choi J Dudley DJ Li C Jenkins SL Myatt L. Placental amino acid transport and placental leptin resistance in pregnancies complicated by maternal obesity. Placenta. (2010) 31:718–24. 10.1016/j.placenta.2010.06.006 20609473

[22] NRC. Nutrient Requirements of Beef Cattle. Washington, DC: National Academies Press (2016).

[23] Livak KJ Schmittgen TD. Analysis of relative gene expression data using real-time quantitative PCR and the 2^Δ^ΔCT method. Methods. (2001) 25:402–8. 10.1006/meth.2001.1262 11846609

[24] Jansson T. Placenta plays a critical role in maternal-fetal resource allocation. Proc Natl Acad Sci USA. (2016) 113:11066–8. 10.1073/pnas.1613437113 27660237 PMC5056066

[25] Ricks RE Cook EK Long NM. Effects of supplementing ruminal-bypass unsaturated fatty acids during late gestation on beef cow and calf serum and colostrum fatty acids, transfer of passive immunity, and cow and calf performance. Appl Anim Sci. (2020) 36:271–84. 10.15232/aas.2019-01900

[26] Micke GC Sullivan TM Soares Magalhaes RJ Rolls PJ Norman ST Perry VEA. Heifer nutrition during early- and mid-pregnancy alters fetal growth trajectory and birth weight. Anim Reprod Sci. (2010) 117:1–10. 10.1016/j.anireprosci.2009.03.010 19394770

[27] Hu R Zou H Wang Z Cao B Peng Q Jing X. Nutritional interventions improved rumen functions and promoted compensatory growth of growth-retarded yaks as revealed by integrated transcripts and microbiome analyses. Front Microbiol. (2019) 10:318. 10.3389/fmicb.2019.00318 30846981 PMC6393393

[28] Chen H Wang C Huasai S Chen A. Effect of prepartum dietary energy density on beef cow energy metabolites, and birth weight and antioxidative capabilities of neonatal calves. Sci Rep. (2022) 12:4828. 10.1038/s41598-022-08809-6 35318381 PMC8941139

[29] Tanner AR Bauer ML Kennedy VC Keomanivong FE Kirsch JD Reynolds LP. Influence of corn supplementation to beef cows during mid- to late-gestation: maternal feed intake, body condition, plasma metabolites, and calf growth. Livest Sci. (2020) 240:104142. 10.1016/j.livsci.2020.104142

[30] Alugongo GM Xiao JX Chung YH Dong SZ Li SL Yoon I. Effects of *Saccharomyces cerevisiae* fermentation products on dairy calves: performance and health. J Dairy Sci. (2017) 100:1189–99. 10.3168/jds.2016-11399 28012624

[31] Fina M Varona L Piedrafita J Casellas J. Sources of sire-specific genetic variance for birth and weaning weight in *Bruna dels Pirineus* beef calves. Animal. (2012) 6:1931–8. 10.1017/S175173111200122X 23031724

[32] Vranković L Aladrović J Ljubić BB Pipal I Prvanović-Babić N Mašek T. Blood biochemical parameters of bone metabolism in cows and calves kept in a beef suckler system during the early postpartum period. Livest Sci. (2018) 211:8–13. 10.1016/j.livsci.2018.02.014

[33] Standiford TJ. Anti-inflammatory cytokines and cytokine antagonists. Curr Pharm Des. (2000) 6:633–49. 10.2174/1381612003400533 10788601

[34] Smith SM Vale WW. The role of the hypothalamic-pituitary-adrenal axis in neuroendocrine responses to stress. Dial Clin Neurosci. (2006) 8:383–95. 10.31887/DCNS.2006.8.4/ssmith 17290797 PMC3181830

[35] Burdick NC Banta JP Neuendorff DA White JC Vann RC Laurenz JC. Interrelationships among growth, endocrine, immune, and temperament variables in neonatal Brahman calves. J Anim Sci. (2009) 87:3202–10. 10.2527/jas.2009-1931 19542503

[36] Cooke RF Bohnert DW. Technical note: bovine acute-phase response after corticotrophin-release hormone challenge. J Anim Sci. (2011) 89:252–7. 10.2527/jas.2010-3131 20870951

[37] Hulbert LE Moisá SJ. Stress, immunity, and the management of calves. J Dairy Sci. (2016) 99:3199–216. 10.3168/jds.2015-10198 26805993

[38] Jin L Yan S Shi B Bao H Gong J Guo X. Effects of vitamin A on the milk performance, antioxidant functions and immune functions of dairy cows. Anim Feed Sci Tech. (2014) 192:15–23. 10.1016/j.anifeedsci.2014.03.003

[39] Wong WY Ward LC Fong CW Yap WN Brown L. Anti-inflammatory γ- and δ-tocotrienols improve cardiovascular, liver and metabolic function in diet-induced obese rats. Eur J Nutr. (2017) 56:133–50. 10.1007/s00394-015-1064-1 26446095

[40] Wang Y Han X Tan Z Kang J Wang Z. Rumen-protected glucose stimulates the insulin-like growth factor system and mTOR/AKT pathway in the endometrium of early postpartum dairy cows. Animals. (2020) 10:357. 10.3390/ani10020357 32102173 PMC7071121

[41] Schaefer AL Krishnamurti CR Heindze AM Gopinath R. Effect of maternal starvation on fetal tissue nucleic acid, plasma amino acid and growth hormone concentration in sheep. Growth. (1984) 48:404–14. 10.1002/mrd.1120090410 6085313

[42] Osorio JS Trevisi E Ballou MA Bertoni G Drackley JK Loor JJ. Effect of the level of maternal energy intake prepartum on immunometabolic markers, polymorphonuclear leukocyte function, and neutrophil gene network expression in neonatal Holstein heifer calves. J Dairy Sci. (2013) 96:3573–87. 10.3168/jds.2012-5759 23587395

[43] Hondares E Iglesias R Giralt A Gonzalez FJ Giralt M Mampel T. Thermogenic activation induces FGF21 expression and release in brown adipose tissue. J Biol Chem. (2011) 286:12983–90. 10.1074/jbc.M110.215889 21317437 PMC3075644

[44] Yan H Xia M Chang X Xu Q Bian H Zeng M. Circulating fibroblast growth factor 21 levels are closely associated with hepatic fat content: a cross-sectional study. PLoS ONE. (2011) 6:e24895. 10.1371/journal.pone.0024895 21949781 PMC3174975

[45] Brett KE Ferraro ZM Yockell-Lelievre J Gruslin A Adamo KB. Maternal-fetal nutrient transport in pregnancy pathologies: the role of the placenta. Int J Mol Sci. (2014) 15:16153–85. 10.3390/ijms150916153 25222554 PMC4200776

[46] Hu C Yang Y Deng M Yang L Shu G Jiang Q. Placentae for low birth weight piglets are vulnerable to oxidative stress, mitochondrial dysfunction, and impaired angiogenesis. Oxid Med Cell Longev. (2020) 2020:8715412. 10.1155/2020/8715412 32566107 PMC7267862

[47] Joshi NP Mane AR Sahay AS Sundrani DP Joshi SR Yajnik CS. Role of placental glucose transporters in determining fetal growth. Reprod Sci. (2021) 29:2744–59. 10.1007/s43032-021-00699-9 34339038

[48] Wooding FBP Fowden AL Bell AW Ehrhardt RA Limesand SW Hay WW. Localisation of glucose transport in the ruminant placenta: implications for sequential use of transporter isoforms. Placenta. (2005) 26:626–40. 10.1016/j.placenta.2004.09.013 16085042

[49] Batistel F Alharthi AS Wang L Parys C Pan YX Cardoso FC. Placentome nutrient transporters and mammalian target of rapamycin signaling proteins are altered by the methionine supply during late gestation in dairy cows and are associated with newborn birth weight. J Nutr. (2017) 147:1640–7. 10.3945/jn.117.251876 28768834

[50] Zhang J Zhang F Didelot X Bruce KD Cagampang FR Vatish M. Maternal high fat diet during pregnancy and lactation alters hepatic expression of insulin like growth factor-2 and key microRNAs in the adult offspring. BMC Genom. (2009) 10:478. 10.1186/1471-2164-10-478 19835573 PMC2770530

[51] Benediktsson R Calder AA Edwards CR Seckl JR. Placental 11 beta-hydroxysteroid dehydrogenase: a key regulator of fetal glucocorticoid exposure. Clin Endocrinol. (1997) 46:161–6. 10.1046/j.1365-2265.1997.1230939.x 9135697

