# Supplementary material for: High energy diet of beef cows during gestation promoted growth performance of calves by improving placental nutrients transport
Source: Front Vet Sci. 2022 Nov 24;9:1053730. doi: 10.3389/fvets.2022.1053730 (PMC9730878; doi:10.3389/fvets.2022.1053730)
Supplement: Supplementary file 1 [file Data_Sheet_1.docx]

Supplementary Material

# Supplementary Table

TABLE S1 Primers used for quantitative real-time PCR of placental samples in this study.

| Genes | Primer sequence (5’-3’) | GenBank ID | Amplicon size, bp |
| --- | --- | --- | --- |
| VEGFA | F: CCCACGAAGTGGTGAAGTTCA | NM_174216.2 | 164 |
|  | R: CCACCAGGGTCTCGATGG |  |  |
| NOS3 | F: GCCAAGCGAGTGAAAGCAA | NM_181037.3 | 194 |
|  | R: GGATCGCCATTCCCAAAG |  |  |
| GLUT1 | F: CCCATCCCATGGTTCATCGT | NM_174602.2 | 136 |
|  | R: CGCACAGTTGCTCCACATAC |  |  |
| GLUT3 | F: CTCGGCCGCGTTCTACTTA | NM_174603.3 | 117 |
|  | R: TCCTCAAAAGTCCTGCCACG |  |  |
| GLUT4 | F: TCTCAGGCATCAATGCGGTT | XM_024977054.1 | 159 |
|  | R: CCACCAAGAACACCGAGACT |  |  |
| SLC38A1 | F: GGAAGGGCGGATACCACTTT | XM_010827702.2 | 168 |
|  | R: TGACACCCCTGTTATCTCAGC |  |  |
| SLC38A2 | F: TGAAAAGCCATTATGCCGATGT | NM_001082424.1 | 148 |
|  | R: CCCACAATCGCATTGCTCAG |  |  |
| SLC38A4 | F: CTGTGCCCATAGTGCTATTC | NM_001205943.1 | 139 |
|  | R: GGCACAAGGATGACCAAA |  |  |
| FATP1 | F: GAGGGCTTCAGGGTCTCTAGGAT | NM_001033625.2 | 199 |
|  | R: CGGAAAGGCCGAAGAGGTCC |  |  |
| FATP4 | F: GCGCTTCATCCGAATCTTTA | NM_001075667.1 | 159 |
|  | R: GGCCACGCTGTTTGAGTAGT |  |  |
| FABP4 | F: TGGTACAAGTACAAAACTGGGAT | NM_174314.2 | 119 |
|  | R: TCTCTCATAAACTCTGGTGGCA |  |  |
| LEP | F: GACACCAAAACCCTCATCAAGAC | NM_173928.2 | 108 |
|  | R: CCCAGGGATGAAGTCCAAACC |  |  |
| IGF-1 | F: ATTACAAAGCTGCCTGCCCC | AF404761 | 248 |
|  | R: ACCTTACCCGTATGAAAGGAATATACGT |  |  |
| IGF-2 | F: CCTCAGCCTCATCCCCTCCTTTGC | AF342811 | 282 |
|  | R: CTGTGCTCTATTTGCTGTGTTGTCT |  |  |
| SOD1 | F: GAGACCTGGGCAATGTG | NM_174615.2 | 126 |
|  | R: CCTAATCTGAACGGAACG |  |  |
| CAT | F: GGCCTCCGCGATCTTT TCAATG | NM_001035386 | 392 |
|  | R: GGGCCGTCACGCTGGTAGTTG |  |  |
| GSH-Px | F: GGGCATCAGGAAAACGCC | NM_174076 | 88 |
|  | R: GCATAAAGTTGGGCTCGAACC |  |  |
| HSP70 | F: TGTCGCTGGGACTGGA | AY149618.1 | 140 |
|  | R: GCCCTCGTACACCTGGAT |  |  |
| 11β-HSD2 | F: CGAGCACTTGAATGGGCAGTT | AF074706 | 147 |
|  | R: CCTGGGTAATAGCGGCGGAGT |  |  |
| GAPDH | F: CGTGTCTGTTGTGGATCTGACCTG | NM_001034034 | 176 |
|  | R: CAACCTGGTCCTCAGTGTAGCCT |  |  |

VEGFA, vascular endothelial growth factor A; NOS3, nitric oxide synthase 3; GLUT1, glucose transporter 1; GLUT3, glucose transporter 3; GLUT4, glucose transporter 4; SLC38A1, amino acid transporter solute carrier family 38 member 1; SLC38A2, amino acid transporter solute carrier family 38 member 2; SLC38A4, amino acid transporter solute carrier family 38 member 4; FATP1, fatty acid transport family protein 1; FATP4, fatty acid transport family protein 4; FABP4, [fatty acid-binding protein](javascript:;) 4; LEP, leptin; IGF-1, [insulin-like growth factor](javascript:;) 1; IGF-2, [insulin-like growth factor](javascript:;) 2; SOD1, superoxide dismutase 1; CAT, catalase; GSH-Px, glutathione peroxidase; HSP70, heat shock protein 70; 11β-HSD2, hydroxysteroid 11-beta dehydrogenase 2; GAPDH, glyceraldehyde-3-phosphate dehydrogenase.

F, forward; R, reverse.
